# Defining intra-tumoral and systemic immune biomarkers for locally advanced head-and-neck cancer – detailed protocol of a prospective, observatory multicenter trial (ImmunBioKHT) and first results of the immunophenotyping of the patients’ peripheral blood

**DOI:** 10.3389/fonc.2024.1451035

**Published:** 2024-09-13

**Authors:** Anna-Jasmina Donaubauer, Benjamin Frey, Manuel Weber, Moritz Allner, Christoph Vogl, Omar Almajali, Lukas Kuczera, Henriette Tamse, Matthias Balk, Sarina Müller, Markus Eckstein, Lilli Zülch, Lia Mogge, Thomas Weissmann, Rainer Fietkau, Marco Kesting, Heinrich Iro, Udo S. Gaipl, Markus Hecht, Antoniu-Oreste Gostian

**Affiliations:** ^1^ Translational Radiobiology, Department of Radiation Oncology, Uniklinikum Erlangen, Friedrich-Alexander-Universität Erlangen-Nürnberg, Erlangen, Germany; ^2^ Department of Radiation Oncology, Uniklinikum Erlangen, Friedrich-Alexander-Universität Erlangen-Nürnberg, Erlangen, Germany; ^3^ Comprehensive Cancer Center Erlangen-EMN, Erlangen, Germany; ^4^ Bavarian Cancer Research Center (BZKF), Erlangen, Germany; ^5^ Department of Oral and Cranio-Maxillofacial Surgery, Uniklinikum Erlangen, Friedrich-Alexander-Universität Erlangen-Nürnberg, Erlangen, Germany; ^6^ Department of Otolaryngology - Head & Neck Surgery, Uniklinikum Erlangen, Friedrich-Alexander-Universität Erlangen-Nürnberg, Erlangen, Germany; ^7^ Institute of Pathology, Uniklinikum Erlangen, Erlangen, Germany; ^8^ Department of Radiotherapy and Radiation Oncology, Saarland University Medical Center, Homburg, Saar, Germany; ^9^ Department of Otorhinolaryngology, Merciful Brothers Hospital St. Elisabeth, Straubing, Germany

**Keywords:** HNSCC, liquid biopsy, immune monitoring, immunological biomarker, prospective clinical trial

## Abstract

The approval and effectiveness of immune checkpoint inhibitors in head-and-neck squamous cell carcinoma (HNSCC) highlights the role of the immune system in this tumor entity. HNSCCs not only interacts with the immune system in the tumor tissue, but also induce systemic effects that may be additionally influenced by further factors such as the microbiome. Nonetheless, reliable immunological biomarkers that predict treatment response and outcome in HNSCC patients are lacking. The currently available biomarkers are mainly limited to analyses from tumor biopsies, while biomarkers from liquid biopsies, such as peripheral blood are not well-established. Thus, the here presented trial aims to identify interactions of intra-tumoral and systemic immune responses and to define prognostic immune signatures. Consequently, not only samples from the tumor tissue, but also from peripheral blood and the microbiome will be studied/are being evaluated and correlated with the clinical outcome. In this prospective, multi-center trial, 1000 HNSCC patients and 100 patients in the control cohort with non-tumor head-and-neck surgery will be enrolled. The local immune status from of the tumor and the microbiome will be sampled before treatment. In addition, the systemic immune status from peripheral blood will be analyzed before and after surgery and after the adjuvant and definitive radio-chemotherapy (RCT). Clinical baseline characteristics and outcome will additionally be collected. Data mining and modelling approaches will finally be applied to identify interactions of local and systemic immune parameters and to define prognostic immune signatures based on the evaluated immune markers. Approval from the institutional review board of the Friedrich-Alexander-Universität Erlangen-Nürnberg was granted in December 2021 (application number 21-440-B). By now, 150 patients have been enrolled in the intervention cohort. The results will be disseminated to the scientific audience and the general public via presentations at conferences and publication in peer-reviewed journals.

## 1 Introduction

Head and neck squamous cell carcinoma (HNSCC) accounts for around 6% of all cancer diseases worldwide (1). In Germany, approximately 50 out of 100,000 inhabitants are diagnosed with HNSCC every year, while men are more likely to be diagnosed than women (2). HNSCC can arise from the mucosa of different localities in the head and neck region, such as the pharynx, larynx, oral cavity or paranasal sinuses and is most commonly caused by tobacco and alcohol consumption or infections with oncogenic strains of the human papilloma virus (HPV) (3). The majority of the patients presents with advanced stage disease. Thus, the therapy for HNSCC often requires multimodal treatment schemes (4).

Immunological biomarkers become increasingly important in the grading and staging, but also in treatment decisions for several tumor diseases, especially in the context of immunotherapy (5, 6). Those biomarkers cover mainly the presence of certain immune cell populations, such as cytotoxic T cells in the tumor microenvironment, as well as the expression of immune checkpoint molecules, such as PD1 and PD-L1 on these immune cells or on the respective tumor cells (6, 7). Recently, immunological biomarkers from peripheral blood receive more attention in cancer therapy, as they may reflect the situation in the tumor tissue. Also, blood-based biomarkers can easily be integrated into the clinical routine and mean less burden for the patients as no repetitive biopsies from the tumor tissue are necessary (8, 9).

In HNSCC, however, immunological biomarkers play only a minor role and predictive biomarkers from peripheral blood remain scarce until today. In the subgroup of oral squamous cell carcinomas (OSCC) however, a transient shift towards postoperative systemic immune tolerance was observed in the peripheral blood compared with patients undergoing minor surgery (10, 11). In addition, increased peripheral blood PD-L1 m-RNA expression in OSCC patients was associated with the occurrence of lymph node metastases and inferior survival (12, 13). Even though immune checkpoint inhibitors (ICIs) have successfully been approved for the therapy of recurrent and metastasized HNSCC, the number of immunological biomarkers that predict the response to immunotherapy is very limited (7, 14). Until today, the expression of PD-L1 on the tumor cells is the only immunological biomarker that is applied for HNSCC in clinical routine, although its predictive value is limited especially as a stand-alone marker (15). In general, the expression of further immune checkpoint molecules on tumor cells, such as TIM-3 or LAG-3 are only insufficiently analyzed in clinical trials for their predictive value. In addition to the local tumor-associated mechanisms, corresponding immunological markers from peripheral blood, such as the frequency of certain cell types have not yet been integrated into the clinical routine, even though promising results have been found in clinical trials (5, 16, 17).

Apart from the direct interactions between tumor and immune cells, it is well-known that the human microbiome is shaping the immune system and is thus also getting into the focus of tumor-immunobiology research. The microbiome can not only impact tumorigenesis, but also the response to immunotherapy. In HNSCC, especially the oral microbiome might be of interest to understand the immunomodulatory processes during HNSCC formation and during (immuno-)therapy (14). Indeed, some studies found a correlation between HNSCC development and certain bacterial strains, but the impact of the microbiome on the therapy of HNSCC, especially on immunotherapy, remains mostly elusive (14, 18–20).

Nowadays, it is evident that singular immunological biomarkers are most likely not sufficient to predict outcome. As the tumor immune response is complex and involves numerous cell types and structures throughout the whole organism, combined immunological scores that involve local immune markers from the tumor tissue and peripheral immune parameters from whole blood (or even including the microbiome composition) should be more promising (5, 17, 21). Such immunological scores are already successfully applied for the classification of colon cancer (22) and could potentially improve the grading and treatment of HNSCC as well and might also predict therapy failure patterns of radiotherapy (RT) in HNSCC (23).

In order to define prognostic immune signatures for HNSCC, the ImmunBio-KHT trial was initiated as interdisciplinary study between ENT-surgery, maxillofacial-surgery and radiation oncology in 2022. This trial aims to set up a heterogeneous, real-world cohort of HNSCC patients undergoing tumor surgery and/or radio-chemotherapy to define prognostic immune matrices. These immune signatures are built from immune markers from the tumor tissue, the peripheral blood, as well as from the microbiome with the objective to create a basis for personalized treatment of HNSCC in the future.

## 2 Methods and analysis

### 2.1 Study design and registration

**Figure 1:**
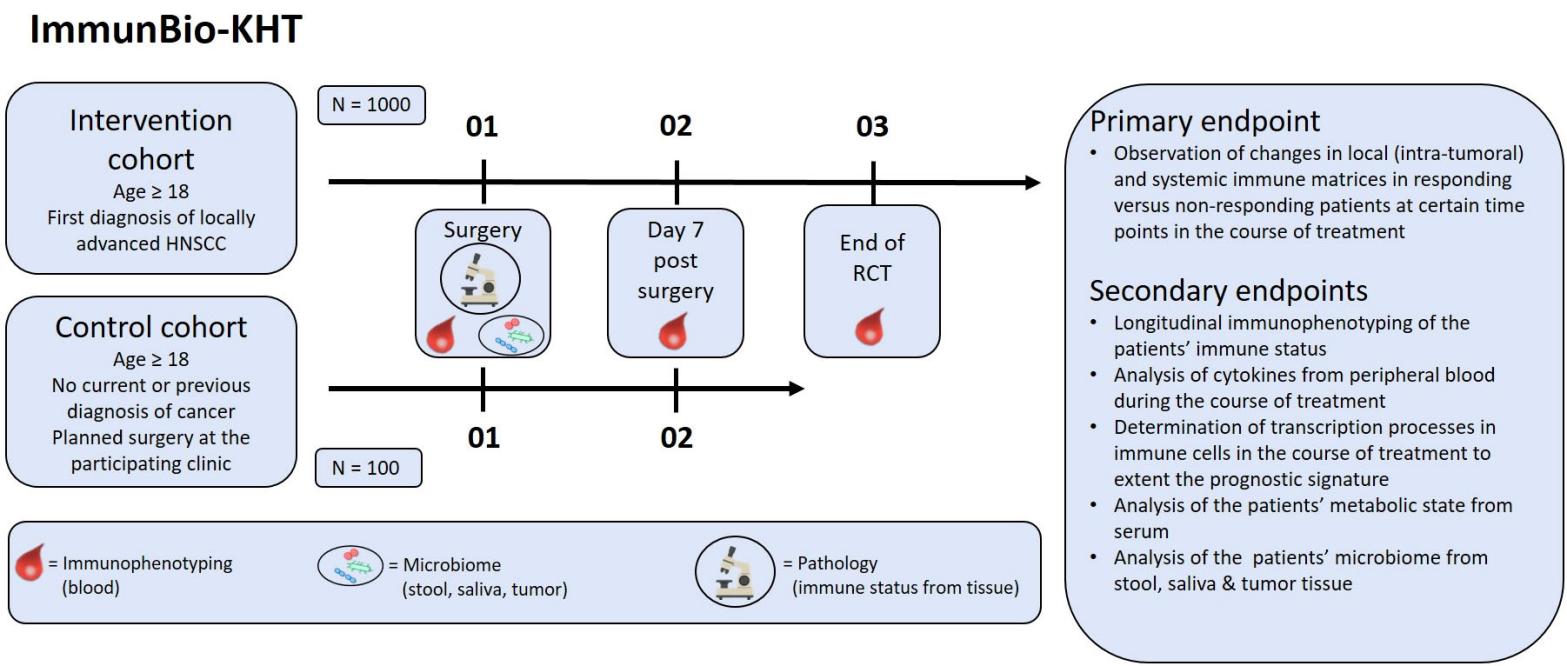
Design of the prospective ImmunBio-KHT trial: In the intervention cohort, 1000 HNSCC patients receiving a tumor surgery and subsequent RCT will be enrolled. In the control cohort, 100 patients with a planned functional surgery in the head-and-neck region will be recruited. Blood for immunophenotyping will be drawn pre-therapeutically, after surgery and after RCT (if applicable). Before surgery the microbiome will be sampled and tissue will be collected from the surgery for pathological examination. The primary endpoint is the determination of changes in immune matrices in responding versus non-responding patients. Further secondary endpoints will be covered in addition.

This prospective, observatory, multicenter trial monitors the local and peripheral immune status and the microbiome of newly diagnosed HNSCC patients (intervention cohort) throughout their treatment. In addition, the equivalent data set of a control cohort will be collected from cancer-free patients that undergo non-cancer surgery of the head-and-neck region. The patients of the intervention cohort undergo ablative surgery and adjuvant RCT or definitive RCT. For the determination of the peripheral immune status, whole blood is taken before surgery, on day seven post-surgery and at the end of RCT. In addition, the oral, tumor and the bowel microbiome is sampled before (stool and saliva samples) and during the surgery (tumor microbiome sample). Also, the local immune status from the tumor tissue is determined after tumor excision by pathological assessment (see **Figure 1**). Oncological data of the patients is followed-up according to the clinical routine or until the death of the patient. In the control cohort, the peripheral immune status is also determined from whole blood before and seven days after surgery. The microbiome is sampled as well (see **Figure 1**).

### 2.2 Recruitment and eligibility criteria

Patients will be recruited from several hospitals in Germany, which include the University Hospitals Erlangen, Regensburg and Augsburg.

In this trial, initially diagnosed HNSCC patients or patients with no current or previous diagnosis of cancer and a planned non-cancer head-and-neck surgery are eligible (see **Figure 1**). The detailed inclusion and exclusion criteria are described in **Table 1**. For the intervention cohort, the key inclusion criteria are (1) age ≥18 years (2), the initial diagnosis of a squamous cell carcinoma of the oral cavity, oropharynx, hypopharynx, larynx or paranasal sinus and (3) consent of the patient for the sampling and preservation of biomaterial. The key exclusion criteria are (1) the occurrence of distant metastases or secondary carcinomas at the time of diagnosis, and (2) carcinomas which do not allow a to take a biopsy without interfering with the pathological evaluation.

**Table 1:** Inclusion and exclusion criteria.

Inclusion and exclusion criteria in the ImmunBio-KHT trial	
Intervention cohort	
**Inclusion**	**Exclusion**
Age ≥ 18 years	Distant metastases and/or simultaneous secondary carcinoma at the time of diagnosis (= inclusion date)
Initial diagnosis of squamous cell carcinoma of the oral cavity, oropharynx, hypopharynx, paranasal sinuses or larynx in stage UICC II-IVB (	Carcinomas in which it is (likely) impossible to take a sample without interfering with the further pathological assessment
Agreement of the patients for sampling blood, saliva and stool as well as consent to the preservation of all samples for further study purposes	Persistent drug abuse
Cognitive ability of the patients to understand the meaning and purpose of the study and agree to it	Patients who are unable or unwilling to behave and receive treatment according to protocol
	Patients who are legally patronized
	Patients who are not eligible for participation in the study due to language barriers
Control cohort	
Age ≥ 18 years	Persistent drug abuse
Diseases other than malignant diseases (patients with the indication for surgery of the ear, nose or maxillofacial surgery)	Patients who are unable or unwilling to behave and receive treatment according to protocol
Absence of a currently existing or previous malignant disease regardless of the anatomical localization	Patients who are legally patronized
Agreement of the patients for sampling blood, saliva and stool as well as consent to the preservation of all samples for further study purposes	Patients who are not eligible for participation in the study due to language barriers
Cognitive ability of the patients to understand the meaning and purpose of the study and agree to it	

The key inclusion criteria for the control cohort are (1) age ≥18 years and (2) the occurrence of diseases with the indication for surgery of the ear or nose or for maxillofacial surgery, which is not a malignant disease. A further key inclusion criterion is (2) the absence of a currently existing or previous malignant disease regardless of the anatomical localization and (3) consent of the patient for the sampling and preservation of biomaterial.

### 2.3 Outcome measures

The peripheral immune status from patients’ blood is determined at the laboratory for Translational Radiobiology, Department of Radiation Oncology of the Uniklinikum Erlangen by a flow cytometry-based, detailed immunophenotyping assay, as already optimized, applied and published elsewhere. This assay allows the discrimination of up to 26 immune cell types from whole blood, along with the quantification of absolute cell counts and the analysis of the expression of several activation markers on the cell surface to determine the activation status (9, 24, 25). Further, serum and plasma are collected to quantify soluble immune mediators, such as cytokines, via multiplex ELISA. Mass spectrometric analyses will be applied to assess the change of metabolites from serum and plasma. In addition, blood is collected into RNA stabilization tubes to generate cell lysates for long-term storage and total RNA isolation from blood cells. Cell pellets generated from whole blood are stored additionally for the isolation of nucleic acids. The sampling and storage of the biomaterial will be conducted by the Central Biobank Erlangen. The transcriptional analyses will be performed via whole exome sequencing, RNA Sequencing, digital droplet PCR or real-time quantitative PCR.

The local immune status from the resected tumor will be determined by analyzing numerous immunological parameters during the pathological examination of the tissue. Those factors include the PD-L1 expression on the tumor cells along with the tumor proportion score (TPS), the combined positive score (CPS), which covers the PD-L1 expression on tumor and immune cells, the tumor area positivity score (TAP) and the tumor-infiltrating lymphocytes (TILs). Further, standard pathological parameters are determined such as the tumor histology and grading, the invasion depth and the growth pattern.

For the analysis of the microbiome, stool, saliva and tumor smear samples are collected before (stool and saliva) and intraoperatively (tumor smear sample). The individual composition of the oral, bowel and tumor microbiome will be determined by metagenomic analyses to detect all non-human species in the samples. The individual qualitative and quantitative diversity of the microbiome will be analyzed by bioinformatical approaches. Here, only the data of the peripheral immune status of the first 150 patients of the intervention cohort will be presented.

### 2.4 Primary endpoint

The primary endpoint is the determination of changes in established immune matrices that are built from the systemic and local (intra-tumoral) immune status at certain time points of evaluation during the course of treatment (see **Figure 1**). Based on the intrinsic immunological biology of the tumors, different immune cells and tumor cell markers will be used to characterize immunological groups by cluster analysis. We will test already validated immune matrices (5) and will further develop and test new immune signatures from the data of intervention and control cohort of the ImmunBio-KHT study.

### 2.5 Secondary endpoints

Several secondary endpoints will be evaluated in the ImmunBio-KHT trial (see **Figure 1**). First, the longitudinal immune status from peripheral blood will be determined by immunophenotyping for the patients of the intervention and the control cohort. In detail, the distribution and amount (cells per milliliter of whole blood) of about 30 immune cell subtypes and certain messenger substances will be examined according to the technique described in (5). Further, serum and plasma of the patients will be used for the quantification of cytokines via multiarray ELISA at certain points during the course of treatment. These analyses will be performed according to the technique described in (5). In addition, transcriptional processes will be determined during the course of treatment in immune cells to extend the prognostic immune signature. Furthermore, the serum and plasma will be used for the mass spectrometric measurement of metabolites, in order to analyze changes in the metabolic state of the patients from baseline to end of radiotherapy. Finally, the individual microbiome of the patients will be analyzed from saliva, stool and tumor smear samples. All the above described analyses (except the analysis of the microbiome) will be conducted before therapy, on day seven after surgery and after RCT.

### 2.6 Sample size calculation

This study is an explorative trial that aims to develop and validate prognostic and predictive immune signatures for HNSCC and will serve as basis for further prospective clinical trials. The sample size calculation was performed by an independent statistician. Based on a previously published immune signature (5) and by choosing a power of 0.95 and a hazard ratio of 0.5, a patient number of n = 100 has been calculated. Still, as the patient collective is heterogeneous and many subgroups are aimed to be evaluated (e.g. based on the different tumor locations, such as oropharynx, larynx, oral cavity and hypopharynx), we decided to increase the patient number to n = 1000. Thereby, a sufficient number of patients per subgroup can be achieved. As the control cohort only serves for comparative analyses on the surgery-induced immune modulation, a patient number of 100 participants was expected to be sufficient. In particular, after reaching the final patient number, many analyses will focus on the comparison of above-mentioned tumor patient subgroups (~100 to 200 patients per subgroup) with the control cohort of 100 patients. Thus, also in regard of these analyses, the planned patient number of 100 in the control cohort was chosen.

### 2.7 Data collection and data analysis

In addition to the biological data sets mentioned in the sections above, baseline clinical characteristics and data on the outcome of the patients are collected. These characteristics include the age, the gender and the p16 status as well as data on substance abuse. Concerning the consumption of alcohol, patients are asked whether they consume alcohol, which kind of alcohol beverages and are asked to quantify the amount of alcohol. In terms of the nicotine abuse, the patients are asked whether they are currently smoking or if the used to smoke in past. Also, the patients are asked to quantify their nicotine consume in pack years. All clinical and biological data sets are collected in a data base that was created using the web-based software REDCap (Research Electronic Data Capture) that allows to build and manage custom data bases for clinical trials. Before analysis, the data will be checked for logic and coherence and a subsequent data cleaning will be performed, if necessary. Interims analyses are planned after the recruitment of 150, 500 and 800 patients, respectively. The project will be accompanied by an independent statistician that is specialized on medical data analysis. In the next step, data mining and data modelling approaches that have already been established in other projects will be applied on the collected data set of the ImmunBio-KHT trial.

The first analysis aims to depict the longitudinal immunological changes of the peripheral immune status in the study cohort, also to clarify that the study is logistically feasible. Further data sets on the local immune status from the tumor tissue, the oncologic data of the patients and the data on the microbiome will be included in following interims analyses. An overview of the biomaterial that has been sampled so far in the ImmunBioKHT trial is presented in **Supplementary Table 1**.

### 2.8 Patient and public involvement

Neither the patients, nor the public was involved in the design, the conduct or the reporting of this trial.

### 2.9 Ethics and dissemination

The approval of the ImmunBio-KHT trial was granted from the institutional review board of the Friedrich-Alexander-Universität Erlangen-Nürnberg on 14th of December 2021 (application number 21-440-B). The trail was also prospectively registered at ClinicalTrial.gov with the registration number: NCT05375266. As the trial is planned as a multicenter study, the Universitätsklinikum Augsburg and the Universitätsklinikum Regensburg plan to collaborate in the ImmunBio-KHT study. The patient enrolment started on April 1st 2022 at the Uniklinikum Erlangen and is planned for the other participating study centers in 2024. The patients will be provided informed consent after a detailed discussion with one of the treating physicians of the study team. The recruitment of 1000 HNSCC patients and 100 patients in the control group is planned to be achieved in March 2026. By the end of March 2027, the evaluation of the endpoints is planned. Subsequently, the results of the ImmunBio-KHT trial will be disseminated to the academic audience as well as to the general public via publication in peer-reviewed journals. In addition, the preliminary and final results will be presented at several scientific conferences.

### 2.10 Composition, roles and responsibilities

All trial processes as well as the data are managed by the study team. The collected biomaterial will be administrated by the Central Biobank Erlangen of the Uniklinikum Erlangen (CeBE) and can be made available for further research projects. The data analysis will be performed by the study team in consultation with an independent statistician that is specialized on medical data analysis.

### 2.11 Protocol modifications

If any protocol modifications need to be performed, the ethics committees, the trial registries as well as the participants will be informed about those changes.

## 3 Results of the first 150 patients

### 3.1 Clinical characteristics

After the recruitment of 150 patients in the study cohort and the first five patients in the control cohort in November 2023 at the University of Erlangen only, a first analysis of the clinical characteristics and the immune status of the patients was performed. The clinical characteristics of the patients have been summarized in **Table 2**.

**Table 2:** Clinical characteristics.

Patient characteristics of the first 150 patients of the ImmunBioKHT trial		
Intervention cohort		
Factor	Category	N
Total number		150
Age (years)	Mean Range	66
		31 - 92
Gender	Male	102
	Female	48
Cancer location	Larynx	27
	Hypopharynx	11
	Oropharynx	44
	Nasopharynx	2
	Oral cavity	64
	Other	2
P16 status	Positive	17
	Negative	27
	No oropharyngeal cancer	106
cT classification	T0	4
	T1	23
	T2	39
	T3	22
	T4	5
	T4a	29
	T4b	3
	N/A	25
cN classification	N0	55
	N1	17
	N2	2
	N2a	5
	N2b	24
	N2c	10
	N3	0
	N3a	0
	N3b	11
	N/A	26
cM classification	M0	96
	M1	11
	Distant metastases expected, cannot be assessed	15
	N/A	28
Smoker’s history	Current smoking	62
	Quit smoking	12
	Never	9
	N/A	67
Alcohol consumption	Yes	32
	No	17
	N/A	101
	Definitive RCT	20
Radio-Chemotherapy	Adjuvant RCT	25
	No RT	57
	N/A	48
Control cohort		
Factor	Category	N
Total number		5
Age	Mean	29
	Range	23 - 35
Gender	Male	2
	Female	3
Performed surgery	Bignathe corrective osteotomy	5
Smoker’s history	Still smoking	0
	Quit smoking	1
	Never	4
	N/A	0
Alcohol consumption	Yes	2
	No	3
	N/A	0

In the intervention cohort, the mean age of the patients was 66 years at study entry, whereas the mean age in the control cohort was 29 years. Thus, at this point, the control cohort is not yet comparable to the intervention cohort in terms of the age. Nonetheless, by further recruitment of patients in the control cohort, this difference will be minimized in the future. In the control cohort, all five patients that have been recruited so far did receive a bignathe corrective osteotomy, as this surgery induces a comparable trauma as the tumor surgery of the HNSCC patients in the intervention cohort (see **Table 2**).

Regarding the intervention cohort, the distribution of the gender of the patients, reflects the normal clinical situation, as around twice as much male patients than female patients have been recruited. In the intervention cohort, the cancer entities of two patients have been summarized as “other” in **Table 2**. One of these patients was diagnosed with thyroid cancer and one patient suffered from a cervical CUP syndrome. The therapy that was applied after the tumor surgery has been assessed as well. For 48 patients however, the therapy has not yet been finished and is thus not yet documented in the affiliated trial data base. Moreover, the TNM classification for the patients of the intervention cohort has been performed. For 25 patients however, the data is not available yet at this time. The HPV testing (p16 status) has only been performed for patients that have been diagnosed with oropharyngeal cancer, as there is no known predictive value of the HPV testing in other HNSCC locations. Finally, as HPV infections are not the only risk factor for HNSCC, but also alcohol and tobacco consumption, the smoker’s history and the consumption of alcohol have been queried in both patient cohorts. However, many patients in the intervention cohort refused to provide information on their nicotine and alcohol consumption. In the control cohort on the other hand, the patient number at this time is too low to compare the noxious behavior to the intervention cohort (see **Table 2**).

### 3.2 Longitudinal modulation of the peripheral immune status

**Figure 2:**
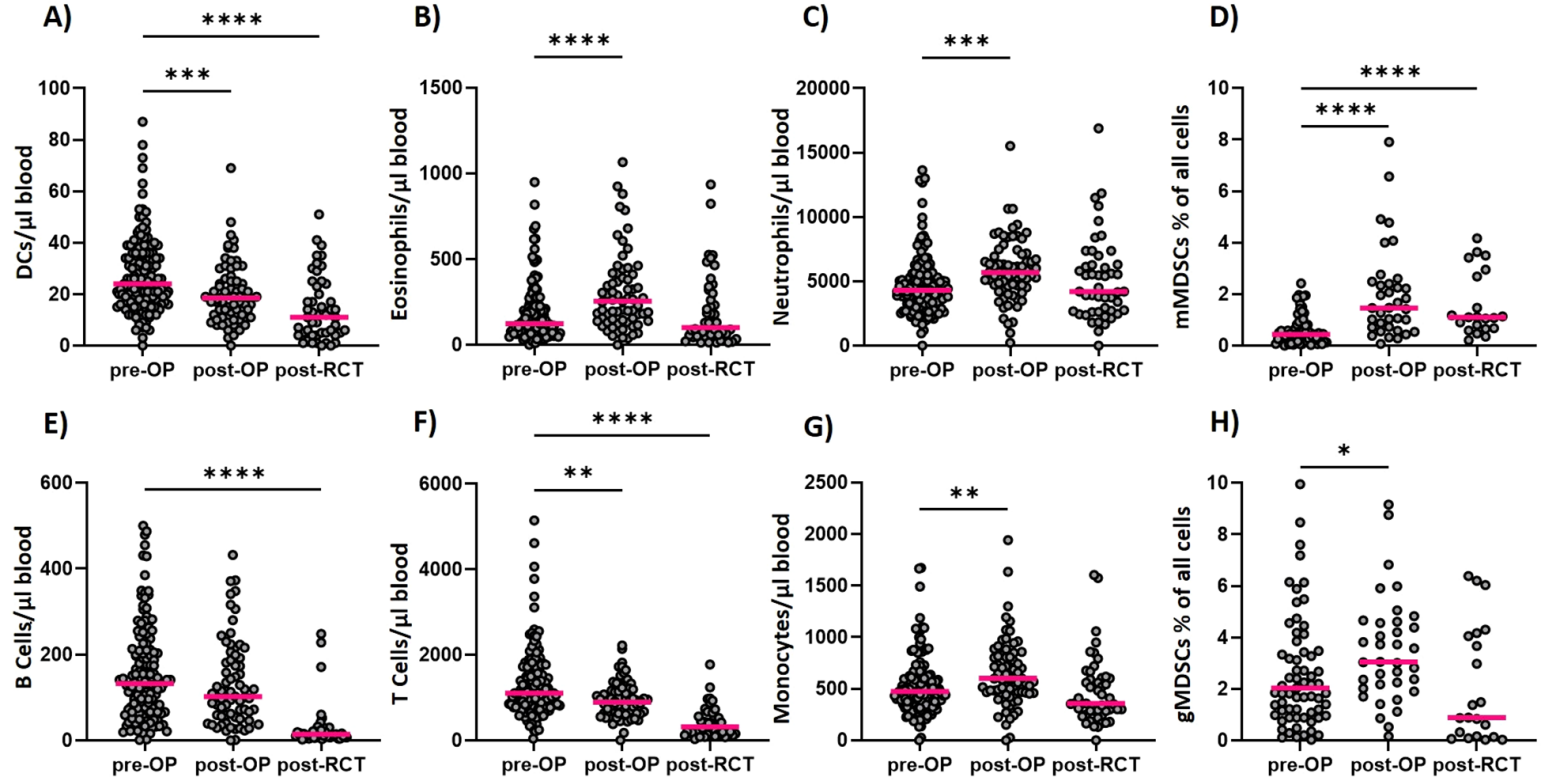
Longitudinal determination of immune cell counts within the ImmunBio-KHT trial: The patients’ systemic immune status was determined from peripheral blood via flow cytometry-based immunophenotyping. Absolute immune cell counts were calculated for all cell types analyzed longitudinally. In **(A)** the counts of DCs over all three time points are depicted. In **(B, C)** the counts of the eosinophilic and neutrophilic granulocytes are depicted, respectively, whereas in **(D)** the counts of mMDSCs and in **(E)** the B cell counts are shown. In **(F)** the T cell counts are depicted and in **(G)** the monocyte and in **(H)** the gMDSC cell counts are shown. The line in each graph shows the median cell count. The Kruskal-Wallis test was applied on all data sets and the significance levels were calculated based on the change from the initial time point (01). (01: n=136, 02: n=56, 03: n=46, *p value ≤ 0.5, ***p value ≤ 0.001, **p<0.01, ****p<0.0001).

**Figure 3:**
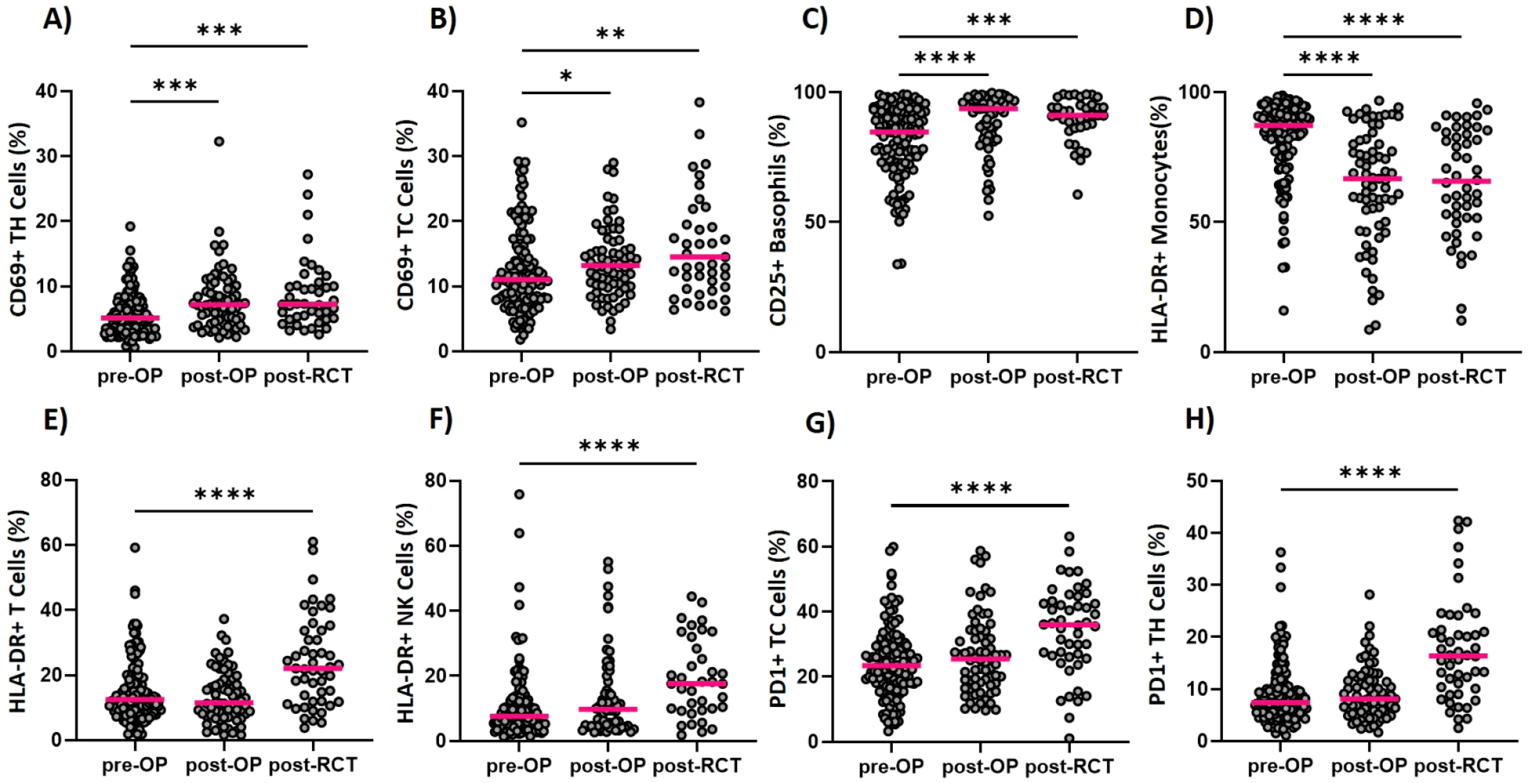
Longitudinal determination of the activation status of immune cells within the ImmunBio-KHT trial: The patients’ immune status was determined from peripheral blood via flow cytometry-based immunophenotyping. The expression of several activation markers was determined and the percentage of positive cells were calculated. Graph **(A)** shows the percentage of CD69 positive T Helper cells and **(B)** of T Killer cells, respectively. The expression of CD25 on basophils is depicted in **(C)**, the expression of HLA-DR on monocytes is shown in **(D)** and the percentage of HLA-DR positive T cells in shown in **(E)**. In **(F)** the HLA-DR expression on NK cells is depicted, while **(G, H)** show the percentage of PD1 positive T Killer and T Helper cells. The line in each graph shows the median percentage of positive cells. The Kruskal-Wallis test was applied on all data sets and the significance levels were calculated based on the change from the initial time point (01). (01: n=136, 02: n=56, 03: n=46, *p value of ≤0.5, **p<0.01, ***p<0.001,****p<0.0001).

The interims analysis reveals that the tumor surgery and the subsequent RCT lead to systemic immune modulations in absolute cell counts of different cell types of the innate and adaptive immune system. Additionally, significant changes in the expression of activation markers on these immune cell types can be detected (see **Figures 2**, **3**). The immunological data presented here is not covering the follow-up data of all patients, as not all patients have completed the follow-up at the time of evaluation and some patients decided to undergo RCT at another clinic and were thus not available for the follow-up time points.

Postoperatively, a significant increase of eosinophilic and neutrophilic granulocytes (**Figures 2B, C**) and of monocytes (**Figure 2G**) has been detected in the peripheral blood. In addition, a significant increase in the percentage of monocytic myeloid-derived suppressor cells (MDSCs) and granulocytic MDSCs has been found (**Figures 2D, H**). The cells of the adaptive immune system, namely the B cells and T cells decreased in the periphery postoperatively (**Figures 2E, F**). A significant decrease in absolute cell counts has also been noticed for the dendritic cells (DCs) on day 7 after surgery (**Figure 2A**). The RCT however, caused a general decline of the immune cell counts in the periphery for all presented immune cell populations. A significant decline has been detected for the DCs (**Figure 2A**), the B cells (**Figure 2E**) and the T cells (**Figure 2F**). For the eosinophils (**Figure 2B**) and the neutrophils (**Figure 2C**), the cell counts generally decreased to the initial level that has been determined before therapy. Even though the cell counts of the monocytic MDSCs (**Figure 2D**) declined after RCT, they were still significantly higher than before surgery. For the granulocytic MDSCs on the other hand, the cell counts declined below the initial median cell count, even though this change was not statistically significant. In sum, a dynamic modulation of the different immune cell subsets has been detected during this therapeutic setting.

The applied therapy had a significant effect not only on the cell counts, but also on the activation status of different immune cell populations (**Figure 3**). A significant upregulation of activation markers has mostly, but not exclusively been detected after RCT on several immune cell populations. On T Helper cells for instance, a significant increase in CD69 expression (**Figure 3A**), but also in PD1 (**Figure 3H**) expression has been detected. For the cytotoxic T cells, the same modulations in the activation state have been detected (**Figures 3B, G**). Furthermore, an increased CD25 expression has been found for the basophilic granulocytes (**Figure 3C**). Even though HLA-DR cannot be considered as a classical activation marker, it is included in our assay since a modulation of the expression of HLA-DR is commonly detected on immune cells. Since HLA-DR is a key molecule in antigen presentation, its differential expression might be relevant in tumor immunology. For T cells and NK cells, an increase in HLA-DR positive cells has been found after RCT (**Figures 3E, F**). On the other hand, HLA-DR positive monocytes were significantly reduced postoperatively but remained unchanged until after the RCT (**Figure 3D**).

## 4 Discussion

The here presented data of the peripheral immune modulation did already prove that the resective and reconstructive tumor surgery and subsequent RCT induces significant immune modulations and alters the frequency and activation of the immune cells that circulate through peripheral blood. More importantly, many of these modulated cell types and activation markers have already been found to be predictive in the context of cancer therapy. Neutrophils for example, have been proven to be prognostically relevant in numerous studies. However, it seems to be dependent on their functionality whether they are favorable for the outcome (5, 26). The HLA-DR expression on monocytes, which was found to be modulated after surgery and RCT in the ImmunBio-KHT patients, is known to be predictive for the response to ICIs in different cancer entities. More precisely, monocytes expressing high levels of HLA-DR predicted a favorable outcome in melanoma, lung cancer and HNSCC patients (5, 27). In line with that, mMDSCs which are characterized by a low expression of HLA-DR are currently investigated for their role in HNSCC in several studies. As MDSCs advance tumors through various mechanisms, their increase in peripheral blood after surgery is not favorable (28). The frequency of circulating cytotoxic T cells has been associated with the response to ICIs not only in HNSCCs, but also in other tumor entities. Particularly meaningful is the expression of activating or inhibiting molecules such as PD1 on these T cells (5, 29, 30). In line with that, we found significant modulations of the activation state of cytotoxic T cells and T Helper cells after RCT in the ImmunBio-KHT cohort. However, the interpretation of the increased expression of activation markers such as CD69 and PD1 on the T cells, is strongly depended on the patient cohort and the applied therapeutic interventions. The increase of PD1+ T cells for example, can be favorable in certain studies, but has also been found to be correlated with a worse prognosis in other trials (5, 29).

Surgery with subsequent RCT is the first-line therapeutic option for locally advanced HNSCC, whereas immunotherapy with ICIs is the first-line treatment in recurrent and/or metastatic HNSCC without curative surgical or radiotherapeutic options (15). Also, in (neo)adjuvant settings, ICIs are currently tested in clinical trials, as well as in different combinatory treatment regimens for HNSCC (4, 31). In the KEYNOTE-412 trial, examining the efficacy of additive pembrolizumab in locally advanced HNSCC patients undergoing definitive RCT, a statistically significant benefit in comparison to HNSCC patients without concomitant Pembrolizumab was almost achieved (32). RCT alone though, is also well-known to modulate the immune system (33), especially the local immune status of the tumor microenvironment. In particular, RT drives the upregulation of immune checkpoint molecules in the tumor microenvironment and alters the frequency of TILs in the tissue (16, 17, 34, 35). Though, it is not surprising that immune related markers for HNSCC have already been proven to be prognostically relevant during R(C)T alone (36–38). In addition, surgical interventions also influence local and systemic immune responses. In OSCC tumor tissue, a shift towards tumor-promoting M2-like macrophages was observed (39). In peripheral blood, among other parameters, a significant downregulation of CD45RO and an upregulation of IL-10 was observed on mRNA level in response to resective and reconstructive surgery (10, 11). Interestingly, the alteration of all analyzed biomarkers was highly dependent on the duration of surgery (10, 11). However, many of the aforementioned immune markers and modulations have not yet been tested in longitudinal settings. Thus, tremendous effort is put in the discovery of immunological biomarkers that are easily integrable into clinical routine and may be determined longitudinally throughout therapy (e.g. liquid biopsies), as many immunological markers change dynamically during therapeutic interventions (5, 17).

The ImmunBio-KHT trial was designed with wide-ranged inclusion criteria that allow the enrollment of a broad HNSCC patient collective that reflects the real-world situation. As HNSCC is a very heterogeneous tumor entity, this real-world patient cohort covers different tumor locations and monitors various confounding factors, such as the p16 status, the smoker’s history or the drinking behavior. With an intended patient number of 1000 in the intervention cohort, the ImmunBio-KHT trial offers the possibility to stratify the patients based on the aforementioned factors. Thereby, biomarkers for specific patient subgroups may be identified and immunological and clinical characteristics of responding and non-responding patients may be described. For HNSCC, numerous biomarker studies are currently running or planned. However, most trials are focusing on very specific treatment schemes or agents or on specific subpopulation of HNSCC patients, such as certain disease stages or particular tumor locations. Thus, the strength of the ImmunBio-KHT study is the fact that it will provide important information on the immune modulation and potential biomarkers during the early disease stages of patients undergoing surgery and/or RCT.

The design of the ImmunBioKHT trial includes a control cohort of patients that undergo surgery for functional diseases in the head-and-neck region without any known malignancy. This cohort fulfills the purpose to understand the changes that are induced by an extensive surgery in the head-and-neck region. Thereby, we aim to discriminate, whether the immunological changes from the pre-surgery to the post-surgery time point are only due to the surgery trauma or if there are effects that can only found in the tumor cohort and may thus be caused by the removal of the tumor and a subsequent change of the immunological milieu. However, we are aware that control cohort is not showing the same clinical features as the tumor patient cohort. Therefore, the control cohort cannot serve the function of determining baseline changes to understand what changes in the immune status of the patients are caused by the tumor itself and which immunological characteristics are due to the remaining clinical features of the individuals, such as the age or the smoking and drinking behavior. However, for this purpose, we can refer to a large data base of immunological data (immunophenotyping of the peripheral immune status) of normal healthy donors without any malignancy that show diverse clinical features and thus might be suitable for further comparative analyses.

Prospective clinical trials are needed for the purpose of biomarker identification and validation. Thus, the primary aim of the ImmunBio-KHT study is the definition of intra-tumoral and systemic immunological biomarkers in newly diagnosed and non-metastatic HNSCC. The ImmunBio-KHT trial will not only focus on the determination of immunological markers from the tumor tissue, but also on liquid biopsies from the peripheral blood. This design is advantageous, as immunological processes in the tumor tissue that may have a high informative and predictive value and can be associated with changes in peripheral immune parameters that are easier to obtain and measure. Thereby, predictive immune signatures from liquid biopsies may be developed, also by making use of Machine Learning-assisted approaches (40).

Apart from the direct measurement of immune cells (either in the tumor or the periphery), the design of the ImmunBio-KHT trial includes the sampling of the oral, tumor and bowel microbiome, as it is becoming ever more evident that the microbiome is not only impacting on the immune system but in consequence as well on immune-related tumoral processes. Different preclinical and clinical investigations have already proven that the microbial composition of the gut microbiome impacts on the response to ICIs in different cancer entities (20, 41–43). For HNSCC, however, only very few studies exist that have analyzed the microbiome of patients. Though, these studies found evidence that the oral microbiome might be involved in HNSCC tumorigenesis (14, 18–20). Based on that finding one can hypothesize that the microbiome might also have further predictive potential in advanced HNSCC. Thus, we aim to analyze the microbial composition in the ImmunBio-KHT trial in a prospective set-up along with the analysis of immunological markers from the tumor tissue and the periphery. These data sets will allow us to create a comprehensive picture of the interaction of the immune system and the microbiome. The interrelation and correlation of the systemic immune status, the local immune status of the tumor microenvironment and the composition of the microbiome has never been analyzed in depth in a large HNSCC cohort. Even without the aim of bridging these data to the patient outcome, the analysis of the microbiome along with the immune status will generate new insights on the immunobiology of HNSCC.

In this first interim analysis of the ImmunBioKHT trial, only the longitudinal data on the peripheral immune status of the first 150 patients of the intervention cohort is presented. Even though predictive and prognostic signatures cannot be built without associating the biological/immunological data with the clinical and oncologic characteristics of the patient cohort, this presentation of the first results proves the feasibility of this large observational trial. In the further course of this trial, the here presented data of the systemic immune status not only needs to be connected to the clinical data, but also to be brought into connection with the local immune status from the tumor tissue. Thereby, we aim to investigate whether the systemic immune status is able to reflect the immunological composition of the tumor micromilieu. The further plans also include the detailed analysis of the patients’ microbiome from stool, saliva and from the tumor tissue as predictive factors for HNSCC. Finally, we plan to connect the biological data sets with the confounding factors and the patients’ outcome.

## Data Availability

The raw data supporting the conclusions of this article will be made available by the authors, without undue reservation.
