# Supplementary material for: Defining intra-tumoral and systemic immune biomarkers for locally advanced head-and-neck cancer – detailed protocol of a prospective, observatory multicenter trial (ImmunBioKHT) and first results of the immunophenotyping of the patients’ peripheral blood
Source: Front Oncol. 2024 Sep 13;14:1451035. doi: 10.3389/fonc.2024.1451035 (PMC11427411; doi:10.3389/fonc.2024.1451035)
Supplement: Supplementary file 1 [file Table1.docx]

**Supplementary Table 1: Biomaterial sampled of the first patients of the ImmunBioKHT trial**

| **Biomaterial sampled so far in the ImmunBioKHT trial** | |
| --- | --- |
| **Intervention cohort** | |
| **Factor** | **N** |
| Total number of patients | 150 |
| **Immunophenotyping samples** |  |
| Time point 1 | 136 |
| Time point 2 | 56 |
| Time point 3 | 46 |
| **Tumor tissue samples** | 150 |
| **Microbiome samples**  Stool  Saliva  Tumor smear |  |
|  | 52 |
|  | 98 |
|  | 57 |
| **Control cohort** |  |
| **Factor** | **N** |
| Total number of patients | 5 |
| **Immunophenotyping samples**  Time point 1 |  |
|  | 5 |
| Time point 2 | 4 |
| **Microbiome samples**  Stool |  |
|  | 2 |
| Saliva | 5 |
